# Redox Mechanisms and NRF2 Signaling in Trigeminal Neuralgia: From Evidence to Network-Guided Prioritization of Candidate Herbal Medicines

**DOI:** 10.3390/antiox15101358

**Published:** 2026-10-20

**Authors:** Jun-Ho Lee, Inkyou Seo, Seung-Ug Hong, Sungyoul Choi

**Affiliations:** 1 College of Korean Medicine, Wonkwang University, Iksan 54538, Republic of Korea; dlwnsgh1319@wku.ac.kr; 2 Department of Digital Healthcare, Graduate School of Jeonbuk Advanced Bio-Convergence Academy (JABA), Wonkwang University, Iksan 54538, Republic of Korea; inca@wku.ac.kr; 3 Department of Korean Medicine Ophthalmology & Otolaryngology & Dermatology, College of Korean Medicine, Dongguk University, Goyang 10326, Republic of Korea; 4 Department of Neuropsychiatry, College of Korean Medicine, Gachon University, Seongnam 13120, Republic of Korea

**Keywords:** trigeminal neuralgia, oxidative stress, NRF2, TRPA1, neuropathic pain, natural products, herbal medicine, echinacoside, network pharmacology

## Abstract

Trigeminal neuralgia is classically explained by neurovascular compression, focal demyelination, and pathological hyperexcitability, but emerging evidence suggests that oxidative stress may form a modifiable molecular layer within this framework. Cerebrospinal fluid from patients contains elevated lipid-peroxidation products capable of activating transient receptor potential ankyrin 1 (TRPA1), a redox-sensitive pain-sensing ion channel, while experimental trigeminal injury studies show that manipulation of nuclear factor erythroid 2-related factor 2 (NRF2) signaling and its downstream effector heme oxygenase-1 can reduce allodynia, oxidative injury, pyroptosis, and satellite glial-cell activation. This review organizes trigeminal redox biology around NRF2-centered antioxidant signaling, in which Kelch-like ECH-associated protein 1 regulates NRF2-dependent transcription through antioxidant response elements, and examines its intersection with TRPA1 and the voltage-gated sodium channel Nav1.7. We then evaluate plant-derived compounds and herbal medicines against this framework. The available evidence remains asymmetric: compounds tested in trigeminal models rarely include causal redox endpoints, whereas compounds with strong NRF2-linked analgesic evidence have largely been studied outside the trigeminal system. A limited multiscale network-pharmacology case study prioritizes Cistanchis Herba as a redox-relevant herbal candidate. Independent pharmacological evidence further identifies its constituent echinacoside as the primary translational compound for trigeminal evaluation, whereas hydroxytyrosol is better regarded as a secondary metabolite-exposure hypothesis. These findings define testable hypotheses rather than established treatments and support experimental evaluation of NRF2-targeted, plant-derived adjunctive strategies in trigeminal neuropathic pain.

## 1. Introduction

Trigeminal neuralgia (TN) is defined by recurrent unilateral facial pain of abrupt onset and termination, limited to the distribution of one or more divisions of the trigeminal nerve and typically triggered by innocuous stimuli such as light touch, chewing, or a draught of air [1,2]. Individual pain attacks are typically brief and severe. TN nevertheless carries a substantial disease burden, with epidemiological studies indicating a considerable population burden and an increased risk of suicide [3,4]. Current classification distinguishes classical TN, attributable to neurovascular compression with morphological changes at the trigeminal root; secondary TN, attributable to an identifiable disease such as multiple sclerosis or a space-occupying lesion; and idiopathic TN [1,2,5]. Beyond this clinical and structural framework, increasing attention has turned to molecular processes that may amplify trigeminal nerve injury and pathological excitability.

The prevailing mechanistic model of classical TN links neurovascular compression at the root entry zone to focal demyelination, altered ion-channel organization, and subsequent ectopic discharge and ephaptic cross-excitation [6,7,8,9]. Accordingly, sodium-channel blockers such as carbamazepine and oxcarbazepine remain first-line treatments, but their long-term use is frequently limited by adverse effects despite high initial efficacy [5,10,11,12]. These limitations motivate interest in complementary mechanisms that could modify the pathological environment underlying trigeminal hyperexcitability. Oxidative and redox signaling may represent such a modifiable layer. Oxidative stress contributes broadly to peripheral neuropathic pain [13,14,15], while trigeminal-specific studies have shown that oxidative products can activate transient receptor potential ankyrin 1 (TRPA1), a redox-sensitive cation channel, on trigeminal nociceptors (pain-sensing sensory neurons) and that cerebrospinal fluid (CSF) from patients with TN contains elevated lipid-peroxidation products capable of activating the TRPA1 channel in TRPA1-expressing cells [16,17]. In the same experimental framework, pharmacological activation of nuclear factor erythroid 2-related factor 2 (NRF2), a transcription factor that controls cellular antioxidant defenses, reversed allodynia after trigeminal nerve injury [17]. Human data therefore support an oxidative abnormality in TN, whereas therapeutic manipulation of NRF2 remains preclinical. The Kelch-like ECH-associated protein 1 (KEAP1)–NRF2–antioxidant response element (ARE) pathway is consequently considered here as a potentially tractable therapeutic axis superimposed on the established compression–demyelination–hyperexcitability framework, rather than as an established causal mechanism of TN.

**Figure 1:**
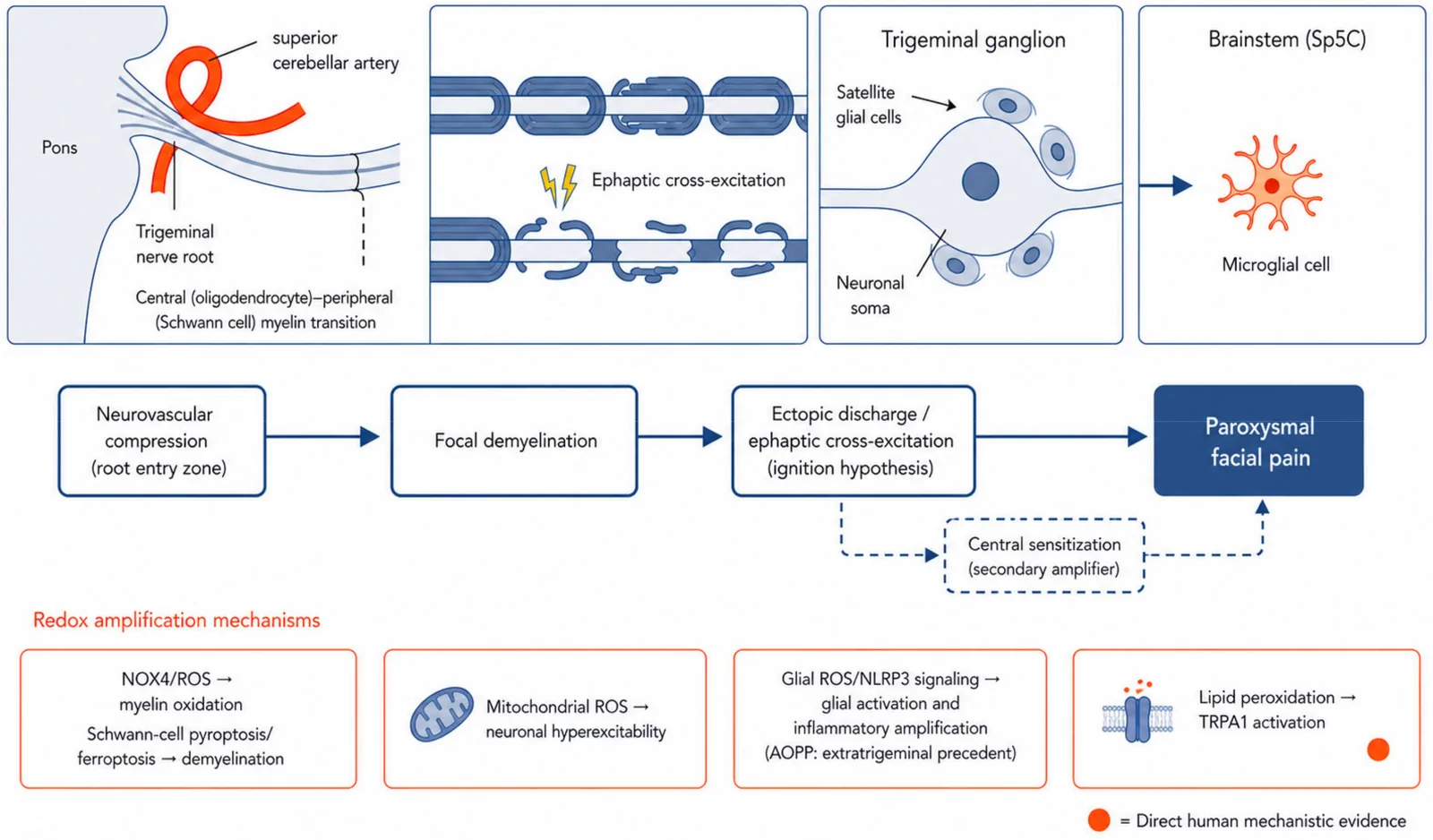
Mechanical and electrical framework of trigeminal neuralgia with redox amplification mechanisms. The core pathway from neurovascular compression to paroxysmal facial pain is shown together with secondary central sensitization and representative redox mechanisms. The orange marker indicates direct human mechanistic evidence for CSF lipid-peroxidation products and TRPA1 activation. The studies supporting each redox mechanism are summarized in Table 1, and these mechanisms are shown converging on NRF2 signaling in Figure 2. Abbreviations: AOPP, advanced oxidation protein products; CSF, cerebrospinal fluid; NLRP3, NOD-like receptor family pyrin domain-containing 3; NOX4, NADPH oxidase 4; ROS, reactive oxygen species; Sp5C, spinal trigeminal nucleus caudalis; TRPA1, transient receptor potential ankyrin 1.

**Figure 2:**
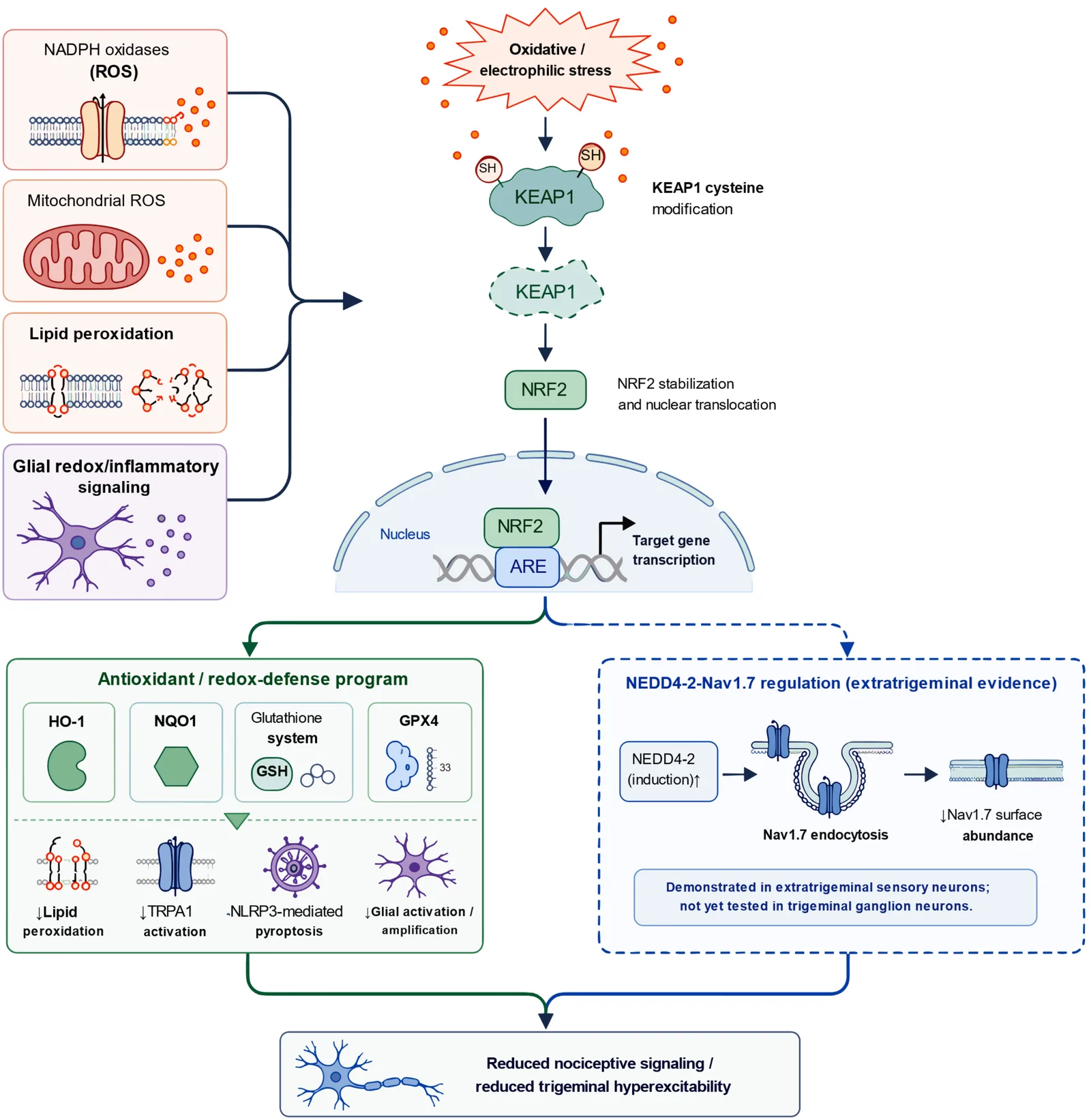
KEAP1–NRF2–ARE signaling as a therapeutic convergence framework in trigeminal redox biology. NRF2 activation coordinates antioxidant/redox-defense responses, while the NRF2–NEDD4-2–Nav1.7 pathway illustrates a potential link to ion-channel regulation demonstrated outside the trigeminal system. The upstream inputs (left) correspond to the redox mechanisms in Figure 1 and Section 2; trigeminal evidence for the downstream outputs is summarized in Table 1, and the plant-derived compounds evaluated against this framework are listed in Table 2. Solid arrows indicate the direction of signaling; the dashed arrow and dashed box denote the NEDD4-2–Nav1.7 branch, which has been demonstrated only in extratrigeminal sensory neurons; downward arrows (↓) indicate a reduction. Abbreviations: ARE, antioxidant response element; GPX4, glutathione peroxidase 4; GSH, glutathione; HO-1, heme oxygenase-1; KEAP1, Kelch-like ECH-associated protein 1; Nav1.7, voltage-gated sodium channel 1.7; NEDD4-2, neural precursor cell expressed developmentally down-regulated 4-2 (an E3 ubiquitin ligase); NLRP3, NOD-like receptor family pyrin domain-containing 3; NQO1, NAD(P)H:quinone oxidoreductase 1; NRF2, nuclear factor erythroid 2-related factor 2; ROS, reactive oxygen species; SH, cysteine thiol group; TRPA1, transient receptor potential ankyrin 1.

**Figure 3:**
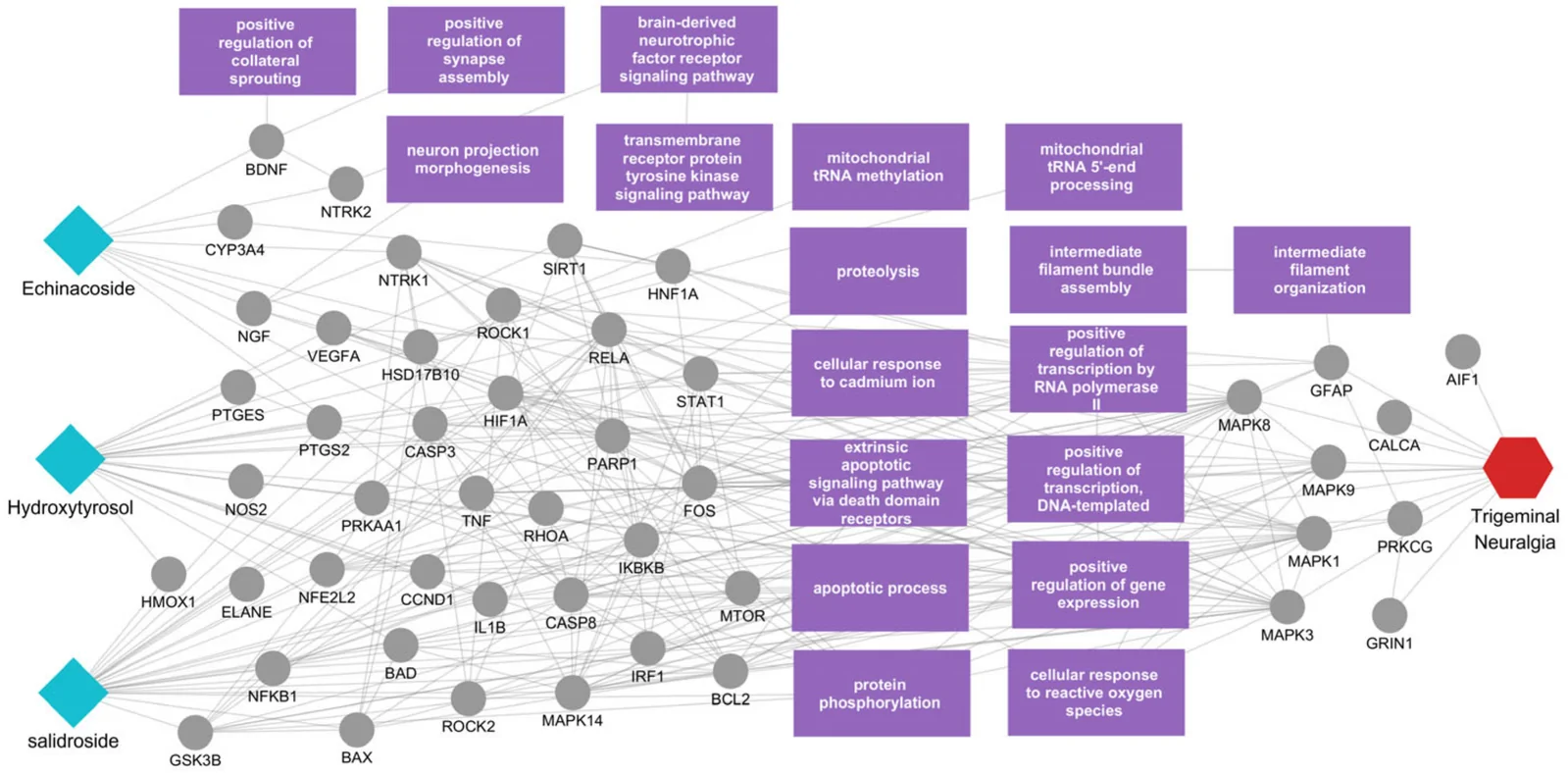
Cistanchis Herba–trigeminal neuralgia subnetwork and candidate interpretation. Hydroxytyrosol represents the network-annotated NFE2L2-linked metabolite/exposure signal, whereas echinacoside is prioritized for experimental testing based on independent pharmacological evidence. Cyan diamonds, annotated compounds/metabolites; gray circles, protein targets (official gene symbols); purple rectangles, enriched Gene Ontology biological-process nodes; red hexagon, trigeminal neuralgia disease node; lines, annotated interactions. Selected gene symbols: NFE2L2, NFE2 like bZIP transcription factor 2 (encoding NRF2); HMOX1, heme oxygenase 1 (encoding HO-1); HIF1A, hypoxia-inducible factor 1 subunit α; NOS2, nitric oxide synthase 2; PARP1, poly(ADP-ribose) polymerase 1; SIRT1, sirtuin 1. Other gene symbols follow official HGNC nomenclature; key network targets are also defined in Table 5 footnote and the Abbreviations section.

In this review, we first summarize the major redox mechanisms operating in the trigeminal system, including generation of reactive oxygen species (ROS), glial redox–inflammatory signaling, lipid peroxidation, and redox-sensitive nociception (Section 2). We then examine the KEAP1–NRF2–ARE pathway as a therapeutic convergence framework and consider its links to trigeminal pain mechanisms and ion-channel regulation (Section 3). Next, we evaluate the current evidence for plant-derived natural products and define the remaining gap between trigeminal efficacy and causal redox/NRF2 validation (Section 4). To address this gap, we apply a limited network-guided analysis to prioritize experimentally testable herbal and compound candidates (Section 5). Finally, we discuss translational considerations and future directions for NRF2-centered strategies in trigeminal neuropathic pain (Section 6). The central question addressed throughout is therefore whether NRF2-directed redox modulation, particularly by plant-derived compounds, represents an experimentally testable therapeutic strategy for TN. Figure 1 and Figure 2 provide the mechanistic framework for this question; Table 1 and Table 2 map the human, preclinical, and natural-product evidence onto that framework; and Table 3, Table 4 and Table 5 and Figure 3 present the network-guided prioritization of candidate herbal medicines and their constituents.

## 2. Redox Mechanisms in the Trigeminal System

Redox processes may intersect with several components of the established compression–demyelination–hyperexcitability framework of trigeminal neuralgia (Figure 1). Experimental evidence indicates that oxidative stress can contribute to myelin injury, glial inflammatory signaling, and nociceptor sensitization, although the anatomical specificity and strength of evidence vary across these mechanisms. In trigeminal injury models, ROS accumulation has been linked to Schwann-cell pyroptosis [20], oxidative stress and satellite glial-cell activation [21], and macrophage-dependent TRPA1 activation [16], while human CSF data provide evidence of increased lipid-peroxidation products capable of activating TRPA1 [17]. Oxidative stress is therefore best regarded as a mechanistically relevant and potentially modifiable layer superimposed on, rather than replacing, the established compression–demyelination–hyperexcitability framework. The following subsections consider the major cellular sources, redox–inflammatory interactions, and nociceptive effectors of this layer. The individual studies supporting each redox mechanism shown in Figure 1 are summarized in Table 1, and the same mechanisms reappear in Figure 2 as upstream inputs that converge on NRF2 signaling.

### 2.1. NADPH Oxidases and Mitochondrial ROS

The NADPH oxidase (NOX) family is a major enzymatic source of ROS that act as signaling molecules in the nervous system, and evidence from peripheral neuropathic-pain models implicates different isoforms in distinct cellular and temporal components of pain processing [13]. NOX2, expressed in neurons, microglia, and infiltrating macrophages, contributes to early oxidative and inflammatory responses after peripheral nerve injury [46,47], whereas NOX4 has been associated with persistent neuropathic pain and oxidative injury to myelin proteins [48]. NOX1 has also been implicated in Schwann-cell-mediated oxidative signaling and the maintenance of neuroinflammation after nerve injury [13]. These isoform-level observations derive predominantly from dorsal-root-ganglion and sciatic-nerve models rather than from the trigeminal system. In the trigeminal ganglion itself, however, ROS accumulate during inflammation, dual oxidase 1 (Duox1) contributes to this response, and psychological stress can further amplify oxidative stress and hyperalgesia [22,23].

Mitochondria constitute another major source of ROS. Both mitochondrial hypofunction and hyperfunction can increase mitochondrial ROS generation in inflammatory and neuropathic pain, and mitochondrial fission mediated by dynamin-related protein 1 (Drp1) may establish a self-amplifying cycle of mitochondrial dysfunction and oxidative stress [14,49]. Direct evidence defining these mitochondrial mechanisms in trigeminal neuralgia remains limited, but peripheral neuropathic-pain models provide a relevant mechanistic precedent. In the sciatic nerve chronic constriction injury (CCI) model, for example, the NRF2 activator RTA-408 (omaveloxolone) attenuated neuropathic pain together with mitochondrial biogenesis associated with peroxisome proliferator-activated receptor-γ coactivator-1α (PGC-1α) [50], suggesting that restoration of mitochondrial homeostasis may offer a more durable strategy than nonspecific scavenging of ROS.

### 2.2. Glial Redox–Inflammatory Mechanisms

Direct trigeminal evidence supports interactions between oxidative stress and glial inflammatory signaling. In a rat model of CCI of the infraorbital nerve (CCI-ION) and in Schwann-like cells exposed to hydrogen peroxide (H_2_O_2_), ROS accumulation was linked to activation of the NOD-like receptor family pyrin domain-containing 3 (NLRP3) inflammasome and pyroptosis (an inflammatory form of programmed cell death), with induction of gasdermin D (GSDMD), interleukin (IL)-1β, and IL-18 [20]. Pharmacological activation of heme oxygenase-1 (HO-1) suppressed these pyroptotic changes in the cellular model, whereas HO-1 inhibition aggravated them [20]. More recently, bone morphogenetic protein 7 (BMP7) was shown to reduce ROS accumulation and satellite glial-cell activation through NRF2/HO-1 signaling in a rat model of CCI of the distal infraorbital nerve (CCI-dION) and in primary satellite glial cells; inhibition of NRF2 with ML385 blocked the cellular protective effect [21]. Together, these findings indicate that redox-sensitive inflammatory signaling operates in more than one glial compartment of the trigeminal system.

Related observations from extratrigeminal models (i.e., models outside the trigeminal system) provide additional mechanistic context. In a sciatic CCI model, advanced oxidation protein products (AOPPs) accumulated in the dorsal root ganglion and activated satellite glial cells through signaling via the receptor for advanced glycation end products (RAGE) and nuclear factor-κB (NF-κB), accompanied by increased ROS and inflammatory cytokine production [51]. The RAGE inhibitor FPS-ZM1 attenuated AOPP-induced satellite glial-cell activation in vitro, whereas chronic AOPP exposure aggravated hyperalgesia and neuroinflammation in vivo [51]. In the Sp5C of a recurrent nitroglycerin-induced migraine model, microglial NLRP3 activation promoted IL-1β release and central sensitization, and NLRP3 blockade attenuated these responses [52]. Although that study did not directly establish ROS as the upstream trigger, broader neuropathic-pain evidence indicates that NOX-derived ROS can contribute to both priming and activation of the NLRP3 inflammasome [15]. These adjacent models therefore support, but do not independently establish, redox–glial mechanisms in TN.

### 2.3. Lipid Peroxidation and Redox-Sensitive Nociception

Lipid peroxidation deserves particular emphasis in TN. Myelin is lipid-rich, making peroxidative injury a plausible link between oxidative stress and demyelination, and lipid peroxidation is also one of the few redox processes for which direct human TN evidence is available. CSF from patients with TN contains elevated 4-hydroxynonenal (4-HNE) and malondialdehyde (MDA), and CSF from most patients, but from few control patients, evoked TRPA1-dependent calcium responses in TRPA1-expressing HEK-293 cells and in mouse trigeminal neurons [17].

Recent experimental evidence extends this relationship from lipid peroxidation to ferroptotic injury (ferroptosis, an iron-dependent form of cell death driven by lipid peroxidation). In a rat CCI-ION model, nerve injury was associated with iron accumulation; reduced expression of glutathione peroxidase 4 (GPX4), the iron exporter ferroportin, and the cystine/glutamate antiporter subunit SLC7A11; Schwann-cell mitochondrial abnormalities; myelin disruption; oxidative stress; and neuroinflammation. Pharmacological inhibition of ferroptosis with ferrostatin-1 or liproxstatin-1 attenuated these changes and reduced demyelination and trigeminal neuropathic pain [53]. These findings complement earlier observations from non-trigeminal neuropathic-pain models in which ferroptosis inhibition reduced pain-related phenotypes [54,55]. GPX4, which reduces lipid hydroperoxides and is regulated within the broader NRF2 antioxidant program, is therefore a particularly relevant node at the intersection of lipid peroxidation and redox defense [56].

TRPA1 is a key redox-sensitive nociceptive transducer that can convert oxidative chemistry into nociceptive signaling. Beyond serving as a marker of lipid peroxidation, 4-HNE is a direct agonist of TRPA1. In a mouse model of trigeminal nerve injury, monocyte and macrophage infiltration generates oxidative stress that activates TRPA1 on trigeminal nociceptors; genetic deletion or pharmacological blockade of TRPA1 abolishes mechanical and cold hypersensitivity, while reducing macrophage recruitment or oxidative burden attenuates both inflammatory infiltration and pain [16]. Within the trigeminal ganglion, ROS also drive TRPA1-dependent hyperalgesia and increase TRPA1 expression, establishing a feed-forward interaction that can be further amplified by stress exposure through Duox1 [22,23].

## 3. The KEAP1–NRF2–ARE Signaling Pathway as a Therapeutic Convergence Axis

### 3.1. Organization of the KEAP1–NRF2–ARE Pathway

NRF2 is maintained at low basal levels by KEAP1, a cysteine-rich adaptor that functions as a homodimer and bridges NRF2 to the Cullin 3 (CUL3)–RING-box protein 1 (RBX1) ubiquitin ligase complex for proteasomal degradation. According to the two-site “hinge-and-latch” model, a KEAP1 homodimer engages a single NRF2 molecule through the high-affinity ETGE motif (the “hinge”) and the lower-affinity DLG motif (the “latch”) within the Neh2 domain of NRF2, with the DLG interaction contributing to the positioning of NRF2 for efficient ubiquitination [57]. Distinct reactive cysteine residues in KEAP1 regulate this process: Cys273 and Cys288 are required for efficient KEAP1-dependent ubiquitination and basal repression of NRF2, whereas Cys151 is required for the inhibition of NRF2 degradation by oxidative stress and electrophilic agents [58]. When KEAP1-mediated degradation is inhibited, newly synthesized NRF2 accumulates and translocates to the nucleus, where it heterodimerizes with small Maf (sMaf) proteins, including MafF, MafG, and MafK; the resulting NRF2–sMaf complex binds AREs to activate target-gene transcription [59]. This transcriptional program includes HO-1 (encoded by the HMOX1 gene), NAD(P)H:quinone oxidoreductase 1 (NQO1), glutathione-synthesis enzymes, and GPX4, among other cytoprotective effectors (Figure 2). Throughout this review, protein names (e.g., NRF2 and HO-1) are used when referring to biological mechanisms, whereas the corresponding official gene symbols (NFE2L2 and HMOX1, respectively) are used when referring to gene-level network annotations (Section 5).

This architecture is relevant to the heterogeneous redox mechanisms described in Section 2. Because KEAP1 functions as a thiol-based sensor of cellular redox stress, NRF2 can coordinate a broad endogenous defense response to oxidative disturbances arising from different cellular sources. The NOX-dependent, mitochondrial, glial, and lipid-peroxidation mechanisms discussed above (Figure 1; Table 1) can therefore be viewed through NRF2 as a common, potentially tractable stress-response framework rather than as a single linear pathway.

Beyond coordinating antioxidant and detoxification genes, NRF2 also influences mitochondrial bioenergetics and quality control, which is directly relevant to the mitochondrial sources of ROS considered in Section 2.1. Genetic loss of Nrf2 has been shown to reduce mitochondrial membrane potential, ATP levels, and respiration, whereas constitutive NRF2 activation increases the availability of substrates for mitochondrial respiration [60]. In a peripheral neuropathic-pain model, the NRF2 activator RTA-408 promoted PGC-1α-associated mitochondrial biogenesis while attenuating neuropathic pain [50]. NRF2 has also been linked directly to mitochondrial dynamics: NRF2 activation following KEAP1 knockdown promoted mitochondrial hyperfusion through enhanced proteasomal degradation of the fission protein Drp1 [61]. In addition, in Drosophila with Parkin or PTEN-induced kinase 1 (Pink1) knockdown, overexpression of the NRF2 ortholog CncC induced mitophagy and reversed neuronal and muscle degeneration phenotypes [62]. Because Drp1-mediated mitochondrial fission can establish a self-amplifying cycle of mitochondrial dysfunction and oxidative stress [49], these findings provide an experimental basis for considering NRF2-dependent mitochondrial regulation as a mechanism extending beyond direct ROS scavenging, although its relevance to trigeminal tissue remains to be established directly.

### 3.2. Evidence from Peripheral Neuropathic Pain

NRF2 activation has shown antinociceptive effects across several peripheral neuropathic-pain models, including diabetic neuropathy, chemotherapy-induced peripheral neuropathy, and nerve-constriction injury [63,64]. Reported mechanisms include induction of HO-1, NQO1, and glutathione-associated defenses, suppression of inflammatory signaling, and improvement of mitochondrial function.

Two pharmacological examples illustrate the translational relevance of this pathway. Dimethyl fumarate, an approved treatment for multiple sclerosis, reduces neuropathic allodynia in rodents through NRF2-dependent signaling [65]. RTA-408, now approved for Friedreich ataxia, attenuated mechanical allodynia and thermal hyperalgesia after CCI while promoting NRF2- and PGC-1α-associated mitochondrial biogenesis [50,66]. These findings establish pharmacological tractability of NRF2 signaling in peripheral neuropathic pain but do not demonstrate efficacy in trigeminal neuralgia.

### 3.3. Trigeminal-Specific Evidence

The trigeminal-specific evidence is recent and increasingly convergent, but the levels of evidence should remain distinct. Human data currently support an oxidative abnormality in TN, whereas direct therapeutic manipulation of NRF2 or related signaling remains preclinical.

Vasavda and colleagues provided the strongest bridge between human observations and experimental NRF2 pharmacology [17]. CSF from patients with TN contained elevated 4-HNE and MDA and evoked calcium responses in HEK-293 cells expressing TRPA1 but not in vector-transfected cells; it also evoked calcium responses in wild-type, but not TRPA1-deficient, mouse trigeminal neurons, whereas control CSF largely did not. In a mouse model of maxillary nerve constriction, pharmacological or genetic activation of NRF2 (sulforaphane or Keap1 deletion) reduced oxidative stress and allodynia to an extent comparable to TRPA1 inhibition, whereas NRF2 deletion aggravated allodynia [17]. A transcriptome-guided drug-repurposing screen further identified exemestane (an aromatase inhibitor) and JQ-1 (an inhibitor of the bromodomain and extra-terminal domain [BET] protein family) as NRF2 network modulators; both induced NRF2 target genes and reduced mechanical and cold allodynia after systemic administration [17]. Exemestane stabilized NRF2, promoted its nuclear translocation, and lost its analgesic effect in NRF2-knockout mice, whereas JQ-1 acted through an unconventional, partly NRF2-independent mechanism and retained some analgesic activity in NRF2-knockout mice [17]. In addition, a single local application of exemestane (25 μg per site) to the constricted maxillary nerve reduced mechanical and cold allodynia, suggesting a local site of action at the nerve, although the clinical relevance of this local delivery route remains unresolved [17].

Additional trigeminal studies provide complementary evidence at downstream components of the NRF2/HO-1 axis. In a rat CCI-ION model and H_2_O_2_-exposed Schwann-like cells, HO-1 activation suppressed ROS-associated NLRP3-mediated pyroptotic changes, whereas HO-1 inhibition aggravated them [20]. In a rat CCI-dION model and primary satellite glial cells, BMP7 reduced oxidative stress and glial activation through NRF2/HO-1 signaling, and the NRF2 inhibitor ML385 blocked the cellular protective effect [21]. Together, these studies support an emerging NRF2/HO-1 therapeutic axis in trigeminal neuropathic pain, while clinical evidence for NRF2-targeted treatment in TN remains absent. The key human and preclinical evidence for redox dysregulation and NRF2-related mechanisms in TN and related trigeminal pain models is summarized in Table 1.

### 3.4. Rationale for NRF2-Centered Therapeutic Screening

NRF2 provides a useful therapeutic screening framework for three reasons. First, it integrates heterogeneous oxidative disturbances into a coordinated endogenous defense program rather than targeting a single ROS source. Second, TN is supported by human evidence for upstream oxidative abnormalities together with multiple preclinical demonstrations that manipulation of NRF2/HO-1 alters trigeminal pain-related phenotypes [17,20,21]. Third, NRF2 signaling may extend beyond antioxidant defense to regulation of neuronal excitability.

Shan and colleagues recently showed that NRF2 activation attenuates neuropathic pain through two experimentally separable mechanisms: restoration of redox homeostasis and suppression of pathologically increased current density of the voltage-gated sodium channel Nav1.7 [39]. The latter effect was mediated indirectly through transcriptional induction of neural precursor cell expressed developmentally down-regulated 4-2 (NEDD4-2), an E3 ubiquitin ligase that tags proteins for removal, which promoted removal of Nav1.7 from the plasma membrane. This mechanism is particularly relevant to TN because current first-line pharmacotherapy already targets voltage-gated sodium-channel excitability, whereas NRF2–NEDD4-2 signaling may regulate channel abundance through a distinct mechanism. However, this pathway was demonstrated in extratrigeminal sensory neurons and has not yet been established in trigeminal ganglion neurons. It should therefore be regarded as a specific translational hypothesis rather than an established mechanism of TN (Figure 2).

## 4. Natural Products: Current Evidence and the Remaining Gap

Surveying the available literature according to three criteria—which pain model was used, whether a redox endpoint was measured, and whether NRF2 involvement was experimentally demonstrated rather than merely discussed—reveals a clear evidence asymmetry. Natural compounds tested in trigeminal or orofacial pain models have generally been evaluated through non-redox mechanisms, whereas compounds with more developed NRF2/redox analgesic evidence have largely been studied in peripheral neuropathic-pain models outside the trigeminal system. This distinction is not absolute, and the underlying search was targeted rather than systematic, but it provides a practical framework for defining the remaining mechanistic gap and prioritizing subsequent experiments.

### 4.1. Compounds with Trigeminal or Orofacial Evidence

Several natural compounds have shown antinociceptive or related effects in trigeminal or orofacial pain models. Resveratrol ameliorated pain-associated phenotypes through neural remodeling associated with cAMP response element-binding protein (CREB)/brain-derived neurotrophic factor (BDNF) signaling and, in a separate study, through AMP-activated protein kinase (AMPK)-dependent suppression of glial activation [25,26]. Curcumin reduced neuropathic pain in a CCI-ION model through suppression of tumor necrosis factor-α (TNF-α) and phosphorylated signal transducer and activator of transcription 3 (pSTAT3) signaling and also improved orofacial allodynia and cognitive impairment in a partial infraorbital nerve transection model [27,28]. Naringenin suppressed hyperexcitability of trigeminal nociceptive neurons in the Sp5C [29].

Importantly, these studies did not establish a causal NRF2 mechanism or incorporate oxidative-stress markers as central mechanistic endpoints. The trigeminal efficacy identified in the literature considered here has therefore been investigated primarily through neurotrophic, inflammatory, kinase, or electrophysiological mechanisms rather than through an explicitly redox-directed experimental design. Doses, routes, and mechanistic endpoints are summarized in Table 2.

### 4.2. Compounds with Redox/NRF2 Evidence Outside the Trigeminal System

More mechanistically developed natural-product redox evidence is found primarily in peripheral neuropathic-pain models outside the trigeminal system. Compounds including (+)-catechin, geraniin, rutin, and quercetin have shown redox-associated antinociceptive effects in such models, with (+)-catechin providing one of the clearer examples of NRF2-linked analgesic activity [30,32,33,34] (Table 2).

Two compounds are particularly relevant to the candidate analysis developed below. Echinacoside had previously shown antinociceptive activity in the sciatic CCI model through inhibition of the P2X7 receptor (P2X7R)/fractalkine (FKN; also known as C-X3-C motif chemokine ligand 1, CX3CL1)/C-X3-C motif chemokine receptor 1 (CX3CR1) pathway [35]. More recently, it was shown to produce NRF2-dependent analgesia and to suppress Nav1.7 through the NRF2–NEDD4-2 pathway in peripheral sensory neurons [39]. Hydroxytyrosol also reduced neuropathic pain together with oxidative and mitochondrial abnormalities in a model of chronic compression of the dorsal root ganglion (CCD), although a causal requirement for NRF2 was not established [40]. Neither compound has yet been evaluated with a causal NRF2-centered design in trigeminal neuropathic pain.

### 4.3. The Remaining Evidence Gap

Taken together, the literature summarized in Table 2 identifies an underexplored intersection between three elements: natural compounds, trigeminal pain models, and experimentally demonstrated redox/NRF2 mechanisms. Compounds already tested in trigeminal models have generally lacked causal NRF2 validation, whereas compounds with stronger NRF2-linked analgesic evidence have largely been studied in sciatic, dorsal-root, or other extratrigeminal models. Because the search was targeted rather than systematic, this should be interpreted as a gap in the indexed literature identified here rather than as a universal absence of evidence.

Adjacent trigeminovascular studies demonstrate that NRF2-directed redox pharmacology can be experimentally examined within trigeminally relevant systems [67,68], although migraine and trigeminal neuralgia should not be conflated. Rather than selecting a familiar antioxidant empirically, we therefore asked whether a disease-centered network could nominate a candidate positioned at this intersection.

## 5. Network Pharmacology-Guided Candidate Prioritization

The preceding sections identify NRF2 as an emerging, preclinically supported therapeutic axis in trigeminal neuropathic pain and define an evidence gap at the intersection of natural products, redox mechanisms, and trigeminal testing. We therefore used a limited network-guided case analysis as a hypothesis-generating step to identify a candidate particularly suitable for experimental follow-up, rather than to infer therapeutic efficacy. Network proximity and functional connectivity should be interpreted as annotation-derived relationships and do not demonstrate target engagement or treatment effects.

The analysis was interpreted in two sequential steps. First, network-based herb-level prioritization was used to identify herbs showing significant overlap with the TN disease set and comparatively strong connectivity to redox/NRF2-related nodes. Second, constituent provenance and independent published pharmacology were considered to determine which chemical entity within the prioritized herb was most appropriate for experimental testing. Thus, the network was used to prioritize the herb and characterize its annotated redox connectivity, whereas final compound selection additionally incorporated chemical provenance and external experimental evidence. The analysis followed the previously described multiscale interactome and gene-set enrichment approaches [44,45,69]; full data sources, parameters, filtering procedures, and correction methods are described in Supplementary Methods S1–S8.

### 5.1. Redox Characteristics of the TN Disease Network

Gene Ontology (GO) enrichment indicated that redox-related biological processes were prominently represented in the trigeminal neuralgia disease set. Among the leading enriched terms were cellular response to ROS (GO:0034614), cellular response to oxidative stress (GO:0034599), and response to ROS (GO:0000302). HMOX1 (the gene encoding HO-1), a canonical NRF2 target, was present in the disease-associated protein set and contributed to multiple oxidative-stress-related terms, whereas NFE2L2 (the gene encoding NRF2) itself was not included in the disease set.

These findings indicate that the TN disease network carries a measurable redox-associated signature even in the absence of direct NFE2L2 annotation. This provided the rationale for examining whether prioritized herbal subnetworks differed in the depth of their connectivity to NRF2-related redox nodes.

### 5.2. Herb-Level Prioritization Identifies Cistanchis Herba

The ten highest-ranking herbs satisfying both diffusion-similarity and target-overlap criteria are summarized in Table 3, and their redox-associated subnetworks are shown in Table 4. All ten prioritized herbs were connected to the disease network through the biological process cellular response to ROS (GO:0034614), indicating that general redox connectivity was common across the candidate set and therefore insufficient by itself to discriminate among herbs.

Differences emerged, however, in the depth of connectivity to the NRF2 axis. Seven herbs contained at most isolated redox-associated proteins, two reached HMOX1, and only one contained an annotated connection to NFE2L2. This herb was Cistanchis Herba. Its disease-focused subnetwork contained NFE2L2 together with HMOX1, SIRT1 (sirtuin 1), NOS2 (nitric oxide synthase 2), HIF1A (hypoxia-inducible factor 1 subunit α), and PARP1 (poly(ADP-ribose) polymerase 1) and connected NFE2L2 to the disease node through MAPK8/MAPK9 (mitogen-activated protein kinases 8 and 9, also known as JNK1/2) within the cellular-response-to-ROS function node. This pattern was not explained simply by subnetwork size, because larger subnetworks did not necessarily extend beyond HMOX1.

Capsici Fructus provided a useful literature-supported reference point within the ranked set. It was the only prioritized herb with established clinical evidence in trigeminal neuralgia through topical capsaicin [42,43] and was therefore treated as a known reference rather than as a novel candidate. Lithospermi Radix, although ranked highest by the network criteria, was not advanced because of pharmacological concerns related to shikonin, including cytotoxicity, limited safety margin, and potential drug interactions [70,71]. Taken together, these results identified Cistanchis Herba as the most informative herb-level candidate for further redox-focused interpretation.

Two herbs are not treated as candidates for reasons outside the network analysis. Capsici Fructus has established clinical evidence in trigeminal neuralgia and is used here as a literature-supported reference point (Section 5.2). Lithospermi Radix, although ranked first, is set aside on pharmacological grounds: its principal constituent shikonin is a necroptosis inducer and pyruvate kinase M2 (PKM2) inhibitor with cytotoxicity toward non-malignant cells, a narrow parenteral safety margin and multiple cytochrome P450 interactions [70], which is difficult to reconcile with an indication requiring sustained dosing in an elderly population. Its novelty is not in question—an aqueous extract reduced mechanical hypersensitivity in oxaliplatin-induced peripheral neuropathy without any oxidative endpoint being measured [71]—and its subnetwork reaches NOS2 and PARP1 but neither NFE2L2 (NRF2) nor HMOX1 (HO-1).

### 5.3. From Network Annotation to Experimental Candidate Selection

Decomposition of the Cistanchis Herba subnetwork revealed an important distinction between network connectivity and experimental candidate selection. Hydroxytyrosol was the only annotated entity carrying an NFE2L2 edge in the curated network and was also connected to HMOX1, SIRT1, NOS2, HIF1A, and PARP1. Salidroside reached several redox-associated nodes, including HMOX1, HIF1A, and PARP1. In contrast, echinacoside showed no redox edge in the curated network and was linked primarily to neurotrophic and signaling targets such as BDNF, nerve growth factor (NGF), and the neurotrophin receptors NTRK1 and NTRK2 (Table 5).

Provenance tracing, however, changed the interpretation of these network annotations. Hydroxytyrosol was identified after oral administration of Cistanche extract rather than as a confirmed free constituent of the crude herb [72]. It can also arise endogenously from dopamine metabolism and has been reported as a metabolite of echinacoside [73,74]. Accordingly, hydroxytyrosol is better interpreted as a Cistanchis Herba-associated metabolite or exposure signal rather than as the primary compound for experimental follow-up.

Echinacoside, by contrast, is a genuine constituent of Cistanche and has independent experimental evidence for NRF2-dependent analgesia and NRF2–NEDD4-2–Nav1.7 regulation in extratrigeminal neuropathic pain [39]. Its prioritization therefore does not arise from an NFE2L2 edge in the curated network, but from combining herb-level network prioritization with constituent provenance and external mechanistic evidence.

Accordingly, echinacoside was selected as the primary translational compound for trigeminal testing, whereas hydroxytyrosol was retained as a secondary metabolite/exposure hypothesis. This distinction yields two experimentally separable questions: whether echinacoside transfers its NRF2-dependent analgesic mechanism to the trigeminal system, and whether hydroxytyrosol-related metabolite exposure contributes to the oral pharmacology of Cistanche-derived phenylethanoid glycosides. These relationships are summarized in Figure 3.

## 6. Translational Considerations and Future Directions

### 6.1. Distinguishing Targeted NRF2 Activation from Untargeted Radical Scavenging

Broad antioxidant supplementation has not consistently improved clinical outcomes and has been associated with harm in some settings, including increased lung cancer incidence in trials of β-carotene-containing regimens and increased mortality in meta-analyses of antioxidant supplementation [75,76,77]. One mechanistic interpretation of these failures is that indiscriminate radical scavenging may interfere with physiological redox signaling without restoring endogenous cellular defense programs. NRF2 activation is conceptually distinct because it induces a coordinated, enzymatically amplified, and transcriptionally regulated cytoprotective response rather than simply increasing reducing capacity [78]. This distinction is important when considering NRF2-directed strategies within the broader concept of “antioxidant therapy.”

### 6.2. Delivery and Target Engagement

Achieving adequate exposure in the trigeminal ganglion and relevant nerve segments may represent an important pharmacokinetic constraint for natural-product interventions. Local application of exemestane to the constricted maxillary nerve reduced allodynia experimentally [17], but the translational relevance of local delivery differs from that of chronic systemic treatment. Echinacoside has demonstrated oral antinociceptive efficacy in peripheral neuropathic pain [39], whereas hydroxytyrosol is orally absorbed but undergoes extensive first-pass metabolism and conjugation in humans [79]. These pharmacokinetic considerations support direct measurement of plasma and trigeminal-tissue exposure rather than assuming target engagement from administered dose alone.

### 6.3. Adjunctive Rather than Substitutive Positioning

NRF2-directed therapy is more appropriately considered as a potential adjunct to, rather than a replacement for, established sodium-channel therapy. Carbamazepine suppresses repetitive firing through state-dependent inhibition of voltage-gated sodium channels, whereas NRF2 activation has been shown in peripheral sensory neurons to reduce pathological Nav1.7 membrane abundance through NEDD4-2-dependent regulation [39]. These mechanisms are potentially complementary, but their combined effects have not been established.

A clinically relevant question is therefore whether NRF2-directed adjunctive treatment could permit dose reduction or improve tolerability of conventional sodium-channel therapy. This is particularly relevant because treatment discontinuation in TN is frequently driven by adverse effects rather than lack of initial efficacy [12]. Potential additivity, dose-sparing, and improvements in tolerability should nevertheless be treated as experimental endpoints rather than anticipated clinical benefits.

### 6.4. Model Limitations

The CCI-ION models widely used in this field produce trigeminal neuropathic pain characterized predominantly by persistent mechanical and cold hypersensitivity. They do not reproduce the defining clinical phenotype of classical TN: brief, paroxysmal, trigger-evoked attacks separated by pain-free intervals [7,9]. Findings from these models should therefore be interpreted as evidence relating primarily to trigeminal neuropathic pain, with extrapolation to classical TN made explicitly and cautiously.

The available human CSF evidence provides an important bridge between these experimental models and clinical TN [17], but the current human evidence remains limited. This distinction should be maintained when interpreting the therapeutic relevance of experimental NRF2-directed interventions.

### 6.5. Future Directions

Future work should first establish the clinical relevance of redox dysregulation in TN through systematic characterization of oxidative-stress biomarkers in patient serum and CSF, ideally including longitudinal sampling before and after microvascular decompression. Such studies could clarify whether the lipid-peroxidation abnormalities currently identified in patients reflect disease activity, treatment response, or a more general state of oxidative stress.

A second priority is tissue- and cell-specific mechanistic validation. Echinacoside should be evaluated in trigeminal neuropathic-pain models with direct assessment of NRF2 dependency and of the NRF2–NEDD4-2–Nav1.7 pathway in trigeminal ganglion neurons. If these effects are confirmed, subsequent studies could examine pharmacokinetic exposure and combination with carbamazepine as a potential dose-sparing strategy. Hydroxytyrosol should remain a secondary metabolite/exposure question rather than a co-equal therapeutic candidate.

More broadly, future studies should clarify how distinct redox-associated injury programs—including pyroptosis and ferroptosis—interact with NRF2 signaling across Schwann cells, satellite glial cells, and trigeminal neurons [20,21,53]. Such experiments could help determine whether these processes represent parallel consequences of oxidative injury or mechanistically interconnected targets within trigeminal neuropathic pain.

### 6.6. Scope and Methodological Limitations

Several limitations of this review should be acknowledged. First, much of the mechanistic rationale—particularly the candidate prioritization in Section 5—rests on computational network analysis and on indirect evidence extrapolated from non-trigeminal models rather than on direct experimental data in trigeminal tissue. The relationships identified here should therefore be regarded as hypotheses that require experimental confirmation, for example, by directly testing NRF2 dependency and the effect of echinacoside on NRF2 activity in trigeminal models (Section 6.5). Second, the underlying literature search was targeted rather than systematic, which may introduce selection bias and omit relevant compounds or mechanisms; the prioritization presented here is accordingly intended as a hypothesis-generating framework rather than a comprehensive or exhaustive ranking. Future work would benefit from a systematic search strategy together with validation across multiple models and complementary data sources.

## 7. Conclusions

Trigeminal neuralgia remains fundamentally a neurovascular compression–demyelination–hyperexcitability disorder. Available human and experimental evidence indicates that oxidative signaling can amplify myelin injury, glial activation, and nociception within this framework. Direct human mechanistic evidence is currently strongest for elevated lipid-peroxidation products in patient cerebrospinal fluid that can evoke TRPA1-dependent responses in vitro, whereas therapeutic manipulation of NRF2 remains preclinical. NRF2 should therefore be considered a mechanistically plausible and experimentally testable therapeutic target for trigeminal neuropathic pain rather than an established treatment mechanism in classical trigeminal neuralgia.

Natural-product evidence is similarly preliminary. The network-guided analysis prioritized Cistanchis Herba at the herb level, while independent pharmacological evidence identified echinacoside as the principal compound for direct trigeminal testing; hydroxytyrosol is better regarded as a secondary metabolite/exposure hypothesis. Overall, the available evidence supports NRF2-centered redox modulation as a testable adjunctive strategy, not as a replacement for established sodium-channel-blocking drugs such as carbamazepine. The immediate next step is to test directly in trigeminal models whether these effects, including those of echinacoside, depend on NRF2.

## Figures and Tables

**Table 1:** Human and experimental evidence for redox alterations in trigeminal neuralgia and related trigeminal pain models.

Study Type	Model or Population	Tissue/Sample	Redox Endpoint Measured	Direction or Finding	Ref
Human	Patients with trigeminal neuralgia vs. controls	CSF	4-HNE; MDA	Elevated in patients; patient CSF evoked TRPA1-dependent calcium responses in HEK-293 cells expressing TRPA1 and in wild-type (but not TRPA1−/−) trigeminal neurons; control CSF largely did not	[17]
Human	Classical TN (*n* = 13) vs. controls (*n* = 13), before and after microvascular decompression	Plasma	NO; CGRP; vitamin D; 2-DE proteome	NO (plasma nitrate/nitrite) elevated in patients and decreased after decompression alongside pain relief, although it remained above control levels; no lipid-peroxidation or antioxidant-capacity assay	[18]
Human	Drug-resistant trigeminal neuralgia, surgical cohort	Serum/CSF	Inflammatory biomarkers	Redox endpoints not reported	[19]
Preclinical	Mouse trigeminal nerve injury (infraorbital constriction)	Trigeminal nerve and ganglion	Macrophage/monocyte-derived oxidative stress; TRPA1 dependency	Oxidative burden increased; TRPA1 deletion or blockade abolished mechanical and cold hypersensitivity	[16]
Preclinical	Mouse chronic constriction of the maxillary branch	Maxillary nerve; trigeminal ganglion	4-HNE, protein carbonylation, MDA; NRF2 expression	Oxidative markers increased after constriction; NRF2 activation (sulforaphane, Keap1 deletion, exemestane) reduced oxidative stress and allodynia comparably to TRPA1 inhibition; NRF2 deletion aggravated allodynia	[17]
Preclinical	Rat CCI-ION; H_2_O_2_-exposed Schwann-like cells	Trigeminal nerve; Schwann cells	ROS; NLRP3, GSDMD, IL-1β, IL-18; HO-1	ROS accumulation upstream of pyroptosis; HO-1 activation suppressed and HO-1 inhibition aggravated it	[20]
Preclinical	Rat CCI-dION; BMP7 intervention	Trigeminal ganglion; satellite glial cells	Oxidative stress; NRF2/HO-1 signaling	Oxidative stress and satellite glial activation reduced via NRF2/HO-1	[21]
Preclinical	Rat inflammatory orofacial hyperalgesia (CFA)	Trigeminal ganglion	Intraganglionic ROS; TRPA1 expression	ROS increased; TRPA1 transcription upregulated, forming a feed-forward loop	[22]
Preclinical	Rat forced-swim stress plus inflammation	Trigeminal ganglion	Oxidative stress; Duox1	Stress amplified inflammation-induced oxidative stress and hyperalgesia	[23]
Preclinical	Rat CCI-ION; tetramethylpyrazine	Trigeminal ganglion	None measured	P2X3 and p38/ERK1/2 pursued; no oxidative endpoint reported	[24]

Abbreviations: 2-DE, two-dimensional gel electrophoresis; 4-HNE, 4-hydroxynonenal; BMP7, bone morphogenetic protein 7; CCI-dION, chronic constriction injury of the distal infraorbital nerve; CCI-ION, chronic constriction injury of the infraorbital nerve; CFA, complete Freund’s adjuvant; CGRP, calcitonin gene-related peptide; CSF, cerebrospinal fluid; Duox1, dual oxidase 1; ERK1/2, extracellular signal-regulated kinases 1 and 2; GSDMD, gasdermin D; H_2_O_2_, hydrogen peroxide; HO-1, heme oxygenase-1; IL, interleukin; MDA, malondialdehyde; NLRP3, NOD-like receptor family pyrin domain-containing 3; NO, nitric oxide; NRF2, nuclear factor erythroid 2-related factor 2; P2X3, P2X purinoceptor 3; p38, p38 mitogen-activated protein kinase; ROS, reactive oxygen species; TN, trigeminal neuralgia; TRPA1, transient receptor potential ankyrin 1.

**Table 2:** Natural compounds evaluated in trigeminal/orofacial and peripheral neuropathic pain models, with redox and NRF2 evidence.

Compound	Model	Dose/Route	Redox Endpoints Measured	NRF2 Implicated	Trigeminal Model	Ref
Resveratrol	Rat CCI-ION (cognitive deficit)	40 mg/kg i.g., twice daily from POD 15	none measured	No (CREB/BDNF)	Yes	[25]
Resveratrol	Rat CCI-ION	p.o., single dose at POD 14 (dose units inconsistently reported in the source)	none measured	No (AMPK)	Yes	[26]
Curcumin	Rat CCI-ION	45 mg/kg i.p., single	none	No (TNF-α/pSTAT3)	Yes	[27]
Curcumin	Mouse partial ION transection	100 mg/kg/day p.o., 14 d	none	No	Yes	[28]
Naringenin	Rat CFA whisker pad, Sp5C recording	50 mg/kg i.p., 2 d	none	No	Yes	[29]
Tetramethylpyrazine	Rat CCI-ION	not stated	none	No (P2X3, p38/ERK)	Yes	[24]
(+)-Catechin	Rat sciatic CCI	50 mg/kg/day p.o. (gavage)	ROS	Yes	No	[30]
(−)-Epicatechin, rutin	Rat sciatic CCI	30 mg/kg p.o.	none	No	No	[31]
Rutin	Rat sciatic CCI	10, 25 mg/kg i.p.	CAT ↑, GSH ↑, nitrite ↓	No	No	[32]
Quercetin	Rat brachial plexus avulsion	25, 50, 100 mg/kg i.g., 7 d	oxidative damage	Not confirmed	No	[33]
Geraniin	Rat sciatic CCI	3–100 mg/kg i.p., 21 d	MDA ↓, GSH ↑, SOD ↑, CAT ↑	Discussed only	No	[34]
Echinacoside	Mouse sciatic CCI	50–200 mg/kg i.p.	none	No (P2X7R/FKN/CX3CR1)	No	[35]
Ursolic acid	Rat sciatic CCI	5, 10, 20 mg/kg p.o.	MDA ↓; protein carbonyl ↓; MPO ↓; NO ↓; total oxidant status ↓; GSH restored	Not confirmed	No	[36]
Kaempferol	Rat sciatic CCI	60 mg/kg/day i.g., 21 d	none	No (TLR4/NF-κB)	No	[37]
Genistein	Mouse sciatic CCI	1–30 mg/kg s.c. (3 mg/kg antinociceptive)	ROS ↓; MDA ↓ in injured paw; antioxidant enzyme activity ↑	No (ERβ)	No	[38]
Echinacoside	Rat / mouse spared nerve injury (SNI)	80 mg/kg/day p.o. × 4 d; 50 μg/kg i.t.	oxidative stress reduced	Yes—dependency established (abolished in NRF2-knockout mice; NEDD4-2/Nav1.7)	No	[39]
Hydroxytyrosol	Rat chronic compression of dorsal root ganglion (CCD)	10 μL of 100 μM, i.t.	mitochondrial ROS ↓; mitochondrial function improved	No causal NRF2 test	No	[40]

Abbreviations: AMPK, AMP-activated protein kinase; BDNF, brain-derived neurotrophic factor; CAT, catalase; CCD, chronic compression of the dorsal root ganglion; CCI, chronic constriction injury; CCI-ION, chronic constriction injury of the infraorbital nerve; CFA, complete Freund’s adjuvant; CREB, cAMP response element-binding protein; CX3CR1, C-X3-C motif chemokine receptor 1; d, days; ERβ, estrogen receptor β; ERK, extracellular signal-regulated kinase; FKN, fractalkine (CX3CL1); GSH, glutathione; i.g., intragastric; i.p., intraperitoneal; i.t., intrathecal; ION, infraorbital nerve; MDA, malondialdehyde; MPO, myeloperoxidase; Nav1.7, voltage-gated sodium channel 1.7; NEDD4-2, neural precursor cell expressed developmentally down-regulated 4-2; NF-κB, nuclear factor-κB; NO, nitric oxide; NRF2, nuclear factor erythroid 2-related factor 2; p.o., per os (oral administration); p38, p38 mitogen-activated protein kinase; P2X3, P2X purinoceptor 3; P2X7R, P2X7 receptor; POD, postoperative day; pSTAT3, phosphorylated signal transducer and activator of transcription 3; ROS, reactive oxygen species; s.c., subcutaneous; SNI, spared nerve injury; SOD, superoxide dismutase; Sp5C, spinal trigeminal nucleus caudalis; TLR4, Toll-like receptor 4; TNF-α, tumor necrosis factor-α. ↑, increased; ↓, decreased.

**Table 3:** Top ten herbs ranked by correlation with the trigeminal neuralgia diffusion profile.

Rank	Herb (Latin)	Correlation †	Overlap (Hypergeometric *p*)	Enrichment	Evidence Status	Ref
1	*Lithospermi Radix*	0.025	5/47 (1.9 × 10^−8^)	7.71	Related evidence only	–
2	*Ganoderma*	0.020	6/50 (2.4 × 10^−10^)	9.62	Related evidence only	[41]
3	*Kalopanacis Cortex*	0.017	3/14 (2.3 × 10^−6^)	5.64	No direct TN evidence	–
4	*Acori Graminei Rhizoma*	0.016	5/47 (1.9 × 10^−8^)	7.71	No direct TN evidence	–
5	*Cistanchis Herba*	0.016	5/50 (2.7 × 10^−8^)	7.58	NRF2 analgesia (non-trigeminal)	[35,39]
6	*Brassicae Campestris Semen*	0.014	4/28 (1.9 × 10^−7^)	6.71	No direct TN evidence	–
7	*Capsici Fructus*	0.013	4/50 (2.1 × 10^−6^)	5.67	Direct TN evidence	[42,43]
8	*Sophorae Tonkinensis Radix Et Rhizoma*	0.012	4/50 (2.1 × 10^−6^)	5.67	No direct TN evidence	–
9	*Morindae Radix*	0.012	4/32 (3.4 × 10^−7^)	6.47	No direct TN evidence	–
10	*Dryopteridis Crassirhizomatis Rhizoma*	0.012	5/50 (2.7 × 10^−8^)	7.58	No direct TN evidence	–

† Correlation between disease and herb diffusion profiles. Overlap *p* values are uncorrected hypergeometric values; Benjamini–Hochberg correction was applied during candidate filtering. Diffusion profiles, overlap, and enrichment were calculated in the present network analysis following the multiscale interactome framework [44,45] (Supplementary Methods S1–S8). Evidence status: No direct TN evidence = no direct trigeminal or neuralgia evidence identified in the targeted search; Related evidence only = related pain or neurological evidence without trigeminal specificity; Direct TN evidence = direct trigeminal-neuralgia evidence; NRF2 analgesia (non-trigeminal) = a constituent has demonstrated NRF2-dependent analgesia in an extratrigeminal model. Abbreviations: NRF2, nuclear factor erythroid 2-related factor 2; TN, trigeminal neuralgia.

**Table 4:** Subnetwork composition and redox content of the ten prioritized herbs, ordered by subnetwork size. Subnetworks were constructed in the present analysis using the multiscale interactome framework [44,45]; compound, target, and biological-function annotations were derived from the databases listed in Supplementary Methods S1.

Herb (Latin)	Annotated Chemical Entities in Subnetwork	Proteins/Functions/Edges	Redox Proteins in Subnetwork	ROS Node
*Dryopteridis Crassirhizomatis Rhizoma*	quercetin, kaempferol, ursolic acid, kaempferol 7-O-α-L-rhamnopyranoside, protocatechuic acid	79/17/697	HMOX1, HIF1A, NOS2, PARP1	Yes
*Capsici Fructus*	capsaicinoids, β-carotene, luteolin-7-O-glucoside, apigenin-7-O-glucoside	70/9/454	HMOX1, HIF1A, NOS2	Yes
*Ganoderma*	genistein, kaempferol, ganoderic acids A/C1/C2/D/F	61/47/515	SIRT1	Yes
*Cistanchis Herba*	salidroside; echinacoside; hydroxytyrosol [post-dose metabolite annotation]	47/18/281	NFE2L2, HMOX1, SIRT1, NOS2, HIF1A, PARP1	Yes
*Lithospermi Radix*	shikonin, isobutylshikonin	33/18/201	NOS2, PARP1	Yes
*Acori Graminei Rhizoma*	α-asarone, β-asarone	32/15/124	—	Yes
*Sophorae Tonkinensis Radix Et Rhizoma*	quercetin, genistein	31/16/257	SIRT1	Yes
*Morindae Radix*	scopoletin	29/8/110	NOS2	Yes
*Brassicae Campestris Semen*	kaempferol 3-O-glucoside	28/8/123	PARP1	Yes
*Kalopanacis Cortex*	kalopanaxsaponin A	23/12/101	NOS2	Yes

The 24-protein redox panel and the definition of “ROS node” are given in Supplementary Methods S5. Proteins/functions/edges, numbers of protein nodes, biological-function nodes, and edges in each herb-specific subnetwork; ROS node, presence of the GO term cellular response to ROS (GO:0034614) in the subnetwork. Abbreviations: GO, Gene Ontology; HIF1A, hypoxia-inducible factor 1 subunit α; HMOX1, heme oxygenase 1 (gene encoding HO-1); NFE2L2, NFE2 like bZIP transcription factor 2 (gene encoding NRF2); NOS2, nitric oxide synthase 2; PARP1, poly(ADP-ribose) polymerase 1; ROS, reactive oxygen species; SIRT1, sirtuin 1.

**Table 5:** Entity-level target assignment within the Cistanchis Herba subnetwork.

Annotated Entity (CID)	Correlation	Overlap (Hypergeometric *p*)	Targets	Redox Targets	Other Principal Targets
Hydroxytyrosol (82755) [absorbed metabolite annotation]	1.39 × 10^−2^	3/21 (3.04 × 10^−6^)	20	NFE2L2, HMOX1, SIRT1, NOS2, HIF1A, PARP1	IRF1, STAT1, PTGS2, VEGFA, TNF, RELA, CASP3, IL1B, PRKAA1
Salidroside (159278)	1.52 × 10^−2^	5/35 (7.75 × 10^−10^)	24	HMOX1, HIF1A, PARP1	BAX, BCL2, BAD, CASP3, ROCK1/2, RHOA, MTOR, GSK3B, NFKB1, RELA, IKBKB, TNF
Echinacoside (5281771)	1.08 × 10^−2^	1/9 (1.30 × 10^−2^)	9	none in curated network; external NRF2 evidence [39]	BDNF, NGF, NTRK1, NTRK2, CASP3, CASP8, MAPK1, CYP3A4, HSD17B10

Network data were generated in the present analysis using the multiscale interactome framework [44,45]; correlation and overlap are defined as in Table 3. CID, PubChem Compound Identifier. Gene symbols follow the official nomenclature of the HUGO Gene Nomenclature Committee (HGNC). Key gene symbols: NFE2L2, NFE2 like bZIP transcription factor 2 (encoding NRF2); HMOX1, heme oxygenase 1 (encoding HO-1); HIF1A, hypoxia-inducible factor 1 subunit α; NOS2, nitric oxide synthase 2; PARP1, poly(ADP-ribose) polymerase 1; SIRT1, sirtuin 1; BDNF, brain-derived neurotrophic factor; NGF, nerve growth factor; NTRK1/NTRK2, neurotrophic receptor tyrosine kinases 1 and 2. Other principal targets: BAD, BCL2 associated agonist of cell death; BAX, BCL2 associated X, apoptosis regulator; BCL2, BCL2 apoptosis regulator; CASP3, caspase 3; CASP8, caspase 8; CYP3A4, cytochrome P450 family 3 subfamily A member 4; GSK3B, glycogen synthase kinase 3β; HSD17B10, hydroxysteroid 17-β dehydrogenase 10; IKBKB, inhibitor of nuclear factor κB kinase subunit β; IL1B, interleukin 1β; IRF1, interferon regulatory factor 1; MAPK1, mitogen-activated protein kinase 1; MTOR, mechanistic target of rapamycin kinase; NFKB1, nuclear factor κB subunit 1; PRKAA1, protein kinase AMP-activated catalytic subunit α1; PTGS2, prostaglandin-endoperoxide synthase 2; RELA, RELA proto-oncogene, NF-κB subunit; RHOA, ras homolog family member A; ROCK1/2, Rho-associated coiled-coil-containing protein kinases 1 and 2; STAT1, signal transducer and activator of transcription 1; TNF, tumor necrosis factor; VEGFA, vascular endothelial growth factor A.

## Data Availability

Source databases used in the network pharmacology analysis are specified in Supplementary Methods S1.

## Supplementary Materials

The following supporting information can be downloaded at: https://www.mdpi.com/article/10.3390/antiox15101358/s1. Supplementary Methods S1–S8: Literature search strategy and network pharmacology pipeline.

## Abbreviations

The following abbreviations are used in this manuscript:

2-DE	two-dimensional gel electrophoresis
4-HNE	4-hydroxynonenal
AMPK	AMP-activated protein kinase
AOPP	advanced oxidation protein product
ARE	antioxidant response element
BDNF	brain-derived neurotrophic factor
BET	bromodomain and extra-terminal domain
BMP7	bone morphogenetic protein 7
CCD	chronic compression of the dorsal root ganglion
CCI	chronic constriction injury
CCI-dION	chronic constriction injury of the distal infraorbital nerve
CCI-ION	chronic constriction injury of the infraorbital nerve
CFA	complete Freund’s adjuvant
CGRP	calcitonin gene-related peptide
CID	PubChem Compound Identifier
CREB	cAMP response element-binding protein
CSF	cerebrospinal fluid
CUL3	Cullin 3
CX3CL1	C-X3-C motif chemokine ligand 1
CX3CR1	C-X3-C motif chemokine receptor 1
Drp1	dynamin-related protein 1
Duox1	dual oxidase 1
ERβ	estrogen receptor β
ERK	extracellular signal-regulated kinase
FKN	fractalkine
GO	Gene Ontology
GPX4	glutathione peroxidase 4
GSDMD	gasdermin D
GSH	glutathione
HGNC	HUGO Gene Nomenclature Committee
HIF1A	hypoxia-inducible factor 1 subunit α
HMOX1	heme oxygenase 1 (gene)
HO-1	heme oxygenase-1 (protein)
i.g.	intragastric
IL	interleukin
ION	infraorbital nerve
i.p.	intraperitoneal
i.t.	intrathecal
JNK	c-Jun N-terminal kinase
KEAP1	Kelch-like ECH-associated protein 1
MAPK	mitogen-activated protein kinase
MDA	malondialdehyde
MPO	myeloperoxidase
Nav1.7	voltage-gated sodium channel 1.7
NEDD4-2	neural precursor cell expressed developmentally down-regulated 4-2
NFE2L2	NFE2 like bZIP transcription factor 2 (gene encoding NRF2)
NF-κB	nuclear factor-κB
NGF	nerve growth factor
NLRP3	NOD-like receptor family pyrin domain-containing 3
NO	nitric oxide
NOS2	nitric oxide synthase 2
NOX	NADPH oxidase
NQO1	NAD(P)H:quinone oxidoreductase 1
NRF2	nuclear factor erythroid 2-related factor 2 (protein)
NTRK	neurotrophic receptor tyrosine kinase
P2X3	P2X purinoceptor 3
P2X7R	P2X7 receptor
PARP1	poly(ADP-ribose) polymerase 1
PGC-1α	peroxisome proliferator-activated receptor-γ coactivator-1α
PKM2	pyruvate kinase M2
p.o.	per os (oral administration)
POD	postoperative day
pSTAT3	phosphorylated signal transducer and activator of transcription 3
RAGE	receptor for advanced glycation end products
RBX1	RING-box protein 1
ROS	reactive oxygen species
s.c.	subcutaneous
SIRT1	sirtuin 1
SLC7A11	solute carrier family 7 member 11 (cystine/glutamate antiporter subunit)
sMaf	small Maf proteins (MafF, MafG, MafK)
SNI	spared nerve injury
SOD	superoxide dismutase
Sp5C	spinal trigeminal nucleus caudalis
TLR4	Toll-like receptor 4
TN	trigeminal neuralgia
TNF-α	tumor necrosis factor-α
TRPA1	transient receptor potential ankyrin 1
