# Supplementary material for: Redox Mechanisms and NRF2 Signaling in Trigeminal Neuralgia: From Evidence to Network-Guided Prioritization of Candidate Herbal Medicines
Source: Antioxidants (Basel). 2026 Oct 20;15(10):1358. doi: 10.3390/antiox15101358 (PMC13701451; doi:10.3390/antiox15101358)
Supplement: Supplementary file 1 [file antioxidants-15-01358-s001.zip › antioxidants-4584499-supplementary.pdf]

Supplementary Methods

Redox Mechanisms and NRF2 Signaling in Trigeminal Neuralgia: From Evidence to Network-Guided Prioritization of Candidate Herbal Medicines

Jun-Ho Lee, Inkyou Seo, Seung-Ug Hong, and Sungyoul Choi

This document provides the literature-search strategy for the narrative review and the full parameterization of the network analysis summarized in Section 5 of the main text. The network analysis follows the framework described previously for hepatic cirrhosis [45], to which the reader is referred for the underlying algorithmic description. Reference numbers correspond to the main-text reference list.

Literature search strategy

A targeted narrative search of PubMed, Web of Science, and Scopus was conducted from database inception through 3 September 2026 using combinations of ("trigeminal neuralgia" OR "trigeminal neuropathic pain" OR "orofacial pain") AND ("oxidative stress" OR "reactive oxygen species" OR "NRF2" OR "antioxidant" OR "lipid peroxidation" OR "ferroptosis"). Compound-specific searches were subsequently performed for candidates discussed in Sections 4 and 5, and reference lists of key primary studies and reviews were screened for additional relevant reports. Preclinical studies were considered when a defined pain model and a quantified behavioral, electrophysiological, biochemical, or molecular endpoint were reported. Because the search was designed to support a mechanistic narrative review rather than a systematic review or meta-analysis, claims of absence are framed as findings of the indexed literature identified by this search rather than universal negatives.

S1. Data sources

Herb–compound annotations. Herb-associated chemical entities were retrieved from the OASIS platform of the Korea Institute of Oriental Medicine, comprising 16,117 annotated compounds across 478 herbs. Herbs with five or fewer annotated chemical entities were excluded, since correlation scores computed on very small annotation sets are unstable.

Compound–target interactions. Annotated entities were merged by PubChem CID with experimentally supported compound–target interactions from DrugBank 5.0, the Therapeutic Target Database, STITCH, and the natural-product interaction set of Huang et al. The top 50 protein targets per herb were retained.

Disease gene set. Trigeminal neuralgia–associated genes (UMLS C0040997) were taken from DisGeNET, restricted to expert-curated associations and excluding associations derived solely from animal models or text mining. After restriction to genes annotated in the enrichment libraries, the analyzed disease set comprised 43 genes.

Provenance caveat. The OASIS compound layer is an annotation resource and does not distinguish crude-drug constituents from post-dose metabolites. Entity provenance was

therefore traced to the primary source before any mechanistic interpretation (Section 5.3).

S2. Network construction and diffusion

Diffusion profiles were computed by biased random walk with restart (restart probability $\alpha = 0.85$) on the multiscale interactome of Ruiz et al. [44], which integrates protein–protein, protein–biological function, and function–function layers. Mechanistic proximity between a herb and the disease was quantified as the cosine correlation between their diffusion profiles.

S3. Herb prioritization

Two criteria were applied jointly:

1. **Diffusion similarity** — cosine correlation between herb and disease diffusion profiles.
2. **Target-set overlap** — hypergeometric test of the overlap between the herb target set and the disease gene set, with Benjamini–Hochberg correction applied across herbs during candidate filtering.

The ten highest-ranking herbs satisfying both criteria were carried forward (Table 3). Table 3 displays the underlying hypergeometric p values rather than the corrected values, for transparency; correction was applied at the filtering stage.

S4. Subnetwork construction

Disease-focused subnetworks were built from the $k = 20$ most visited nodes per herb–disease pair. Subnetwork composition is reported in Table 4 as proteins / biological functions / edges.

S5. Redox panel screening

Subnetwork node lists were screened against a predefined panel of 24 redox-associated proteins:

NFE2L2, KEAP1, HMOX1, NQO1, GCLC, GCLM, SOD1, SOD2, CAT, GPX1, GPX4, SLC7A11, TXN, PRDX1, ALOX5, CYP1B1, NOS2, NOS3, HIF1A, PARP1, SIRT1, XDH, CYBB, NOX4

TRPA1 and SCN9A were additionally tracked as redox-sensitive nociceptive effectors. The panel was defined before subnetwork inspection. "ROS node" in Table 4 denotes the presence of *cellular response to reactive oxygen species* (GO:0034614) as a biological-function node bridging annotated entities to the disease node.

S6. Entity-level edge decomposition

Subnetwork edge lists were decomposed at the level of individual annotated entities to identify which compound–protein edges account for each redox connection (Table 5). This step distinguishes entity-level assignments from herb-level connectivity and is the basis for the two-stage candidate logic described in Section 5.

An important asymmetry applies to the interpretation of these edges. The presence of an edge is a statement about curated interaction records; the absence of an edge is not evidence that an interaction does not exist, since curated resources lag primary publication. This is directly relevant to echinacoside, whose NRF2-dependent analgesic activity was reported after the curated resources used here were assembled.

S7. Functional enrichment

Enrichment was performed in Enrichr [69] against Gene Ontology (Biological Process), with an adjusted $p < 0.05$ threshold.

S8. Scope and limitations

The analysis is a hypothesis-generating prioritization step and is not presented as an original network-pharmacology study. Network proximity and functional connectivity are annotation-derived relationships; they do not demonstrate target engagement or therapeutic efficacy. Predicted connectivity was not experimentally validated in this work.
